# Supplementary material for: Causal Relationship between Aging and Anorexia Nervosa: A White-Matter-Microstructure-Mediated Mendelian Randomization Analysis
Source: Biomedicines. 2024 Aug 16;12(8):1874. doi: 10.3390/biomedicines12081874 (PMC11351342; doi:10.3390/biomedicines12081874)
Supplement: Supplementary file 1 [file biomedicines-12-01874-s001.zip › Supplementary Figure/Figure S4/Singe SNP plot of MR analysis for TL to ubm-b-1458.pdf]

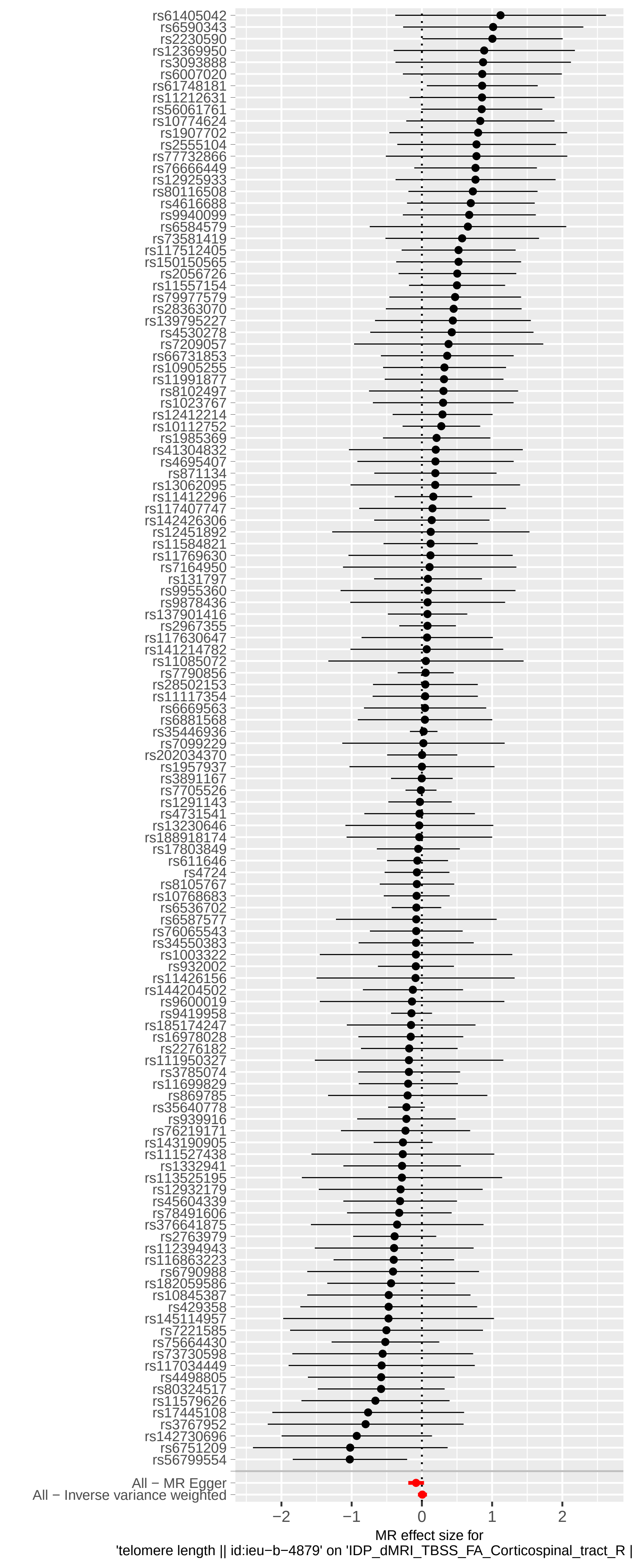
rs61405042
rs6590343
rs2230590
rs12369950
rs3093888
rs6007020
rs61748181
rs11212631
rs56061761
rs10774624
rs1907702
rs2555104
rs77732866
rs76666449
rs12925933
rs80116508
rs4616688
rs9940099
rs6584579
rs73581419
rs117512405
rs150150565
rs2056726
rs11557154
rs79977579
rs28363070
rs139795227
rs4530278
rs7209057
rs66731853
rs10905255
rs11991877
rs8102497
rs1023767
rs12412214
rs10112752
rs1985369
rs41304832
rs4695407
rs871134
rs13062095
rs11412296
rs117407747
rs142426306
rs12451892
rs11584821
rs11769630
rs7164950
rs131797
rs9955360
rs9878436
rs137901416
rs2967355
rs117630647
rs141214782
rs11085072
rs7790856
rs28502153
rs11117354
rs6669563
rs6881568
rs35446936
rs7099229
rs202034370
rs1957937
rs3891167
rs7705526
rs1291143
rs4731541
rs13230646
rs188918174
rs17803849
rs611646
rs4724
rs8105767
rs10768683
rs6536702
rs6587577
rs76065543
rs34550383
rs1003322
rs932002
rs11426156
rs144204502
rs9600019
rs9419958
rs185174247
rs16978028
rs2276182
rs111950327
rs3785074
rs11699829
rs869785
rs35640778
rs939916
rs76219171
rs143190905
rs111527438
rs1332941
rs113525195
rs12932179
rs45604339
rs78491606
rs376641875
rs2763979
rs112394943
rs116863223
rs6790988
rs182059586
rs10845387
rs429358
rs145114957
rs7221585
rs75664430
rs73730598
rs117034449
rs4498805
rs80324517
rs11579626
rs17445108
rs3767952
rs142730696
rs6751209
rs56799554
All − MR Egger
All − Inverse variance weighted
−2
−1
0
1
2
MR effect size for
'telomere length || id:ieu−b−4879' on 'IDP_dMRI_TBSS_FA_Corticospinal_tract_R |
